# Body Mass Index Is a Marker of Nutrition Preparation Sufficiency Before Surgery for Crohn's Disease From the Perspective of Intra-Abdominal Septic Complications: A Retrospective Cohort Study: Erratum

**DOI:** 10.1097/01.md.0000499793.71093.bb

**Published:** 2015-10-30

In the article “Body Mass Index Is a Marker of Nutrition Preparation Sufficiency Before Surgery for Crohn's Disease From the Perspective of Intra-Abdominal Septic Complications: A Retrospective Cohort Study”,^1^ which appeared in Volume 94, Issue 35 of *Medicine*, some information in the affiliation section was originally presented incorrectly. The article has since been corrected online.
